# A study comparing positive benefits for parents, and their children, of children attending the UK’s holiday activities and food program to parents of non-attendees

**DOI:** 10.3389/fpubh.2025.1474400

**Published:** 2025-02-25

**Authors:** Margaret Anne Defeyter, Paul B. Stretesky, Gillian V. Pepper

**Affiliations:** ^1^Department of Social Work, Education and Community Wellbeing, Northumbria University, Newcastle upon Tyne, United Kingdom; ^2^School of Social and Political Sciences, University of Lincoln, Lincoln, England, United Kingdom; ^3^Department of Social Sciences, Northumbria University, Newcastle upon Tyne, United Kingdom; ^4^Department of Psychology, Northumbria University, Newcastle upon Tyne, United Kingdom

**Keywords:** holiday activities and food, physical activity, food insecurity, school holidays camps, parental wellbeing

## Abstract

The Holiday Activities and Food (HAF) is a UK Department for Education (DfE) funded program that provides free food and activities for 5–16-year-olds in receipt of means-tested free school meals. This evaluation focuses on parent/caregiver perceptions of HAF benefits during the 2021 and 2022 school holidays for a sample of parents/caregivers whose children attended HAF (*n* = 736) and a sample who did not attend HAF (*n* = 885). The results show that parents of children who attend HAF for 4 weeks (i.e., the ‘4-Week’ HAF treatment group) report that their children engage in more weeks of physical activity compared to children in the Non-Attendee group (*b* = 0.59, 95% CI [0.25, 0.94]). Parents/caregivers of children who attended HAF for 6 weeks or more report no significant difference in household food insecurity compared to parents/caregivers in the Non-Attendee group (*b* = −0.27, 95% CI [−0.70, 0.16]). The results also show that parents/caregivers are *more* concerned about affordable childcare if their children attend 6 weeks or more of HAF (*b* = −1.33, 95% CI [−2.07, −0.59]). For parents and caregivers of children who attend HAF for 1 to 5 weeks there is no difference in self-reported *Parental Wellbeing* compared to parents/caregivers of non-attending children (*b* = 0.57, 95% CI [−0.09, 1.23]), but parents/caregivers whose children attend 6 weeks or more of HAF report significantly better wellbeing than parents in the control group (*b* = 1.12, 95% CI [0.56, 1.69]). Parents and caregivers of attendees in the HAF treatment groups are no more or less likely to believe that children are safe in their neighborhood than in the Non- Attendee group (*b* = 0.12, 95% CI [−0.11, 0.34] for 6 or more weeks of attendance vs. non-attendees). These findings are discussed in relation to prior research, and we make several HAF policy recommendations.

## An evaluation of holiday activities and food

The Holiday Activities and Food (HAF) Program is a UK Department for Education (DfE) funded (≈ £200 m p.a.) program rolled out across all higher tier local authorities in England in 2021. HAF provides free food and enriching activities for 5–16-year-olds enrolled for free school meals, and financial, health and wellbeing advice and signposting for parents and carers (1). Each local authority has a discretionary budget that can be used to offer HAF places to children identified as requiring support during the holidays, but who are not in receipt of FSM. Activities are normally based in holiday clubs that operate from a variety of venues (e.g., schools, community centers) to ensure that children have access to nutritious meals and enriching physical and cultural activities in a safe environment, especially while their parents and caregivers are working and/or engaged in other activities (2, 3).

While the nationwide HAF program has been operating for three years there have been no peer-reviewed, quantitative evaluations of HAF that have compared parents’ perceptions of physical activity, household food insecurity, affordable childcare, wellbeing and safety for non-HAF attendees and HAF attendees, according to their level of attendance in HAF. The present evaluation addresses the absence of research on the effectiveness of HAF by analyzing data obtained from 1,650 parents/caregivers in a large UK metropolitan area in 2021 and 2022. We do this by focusing on parent/caregivers and determining whether those with children enrolled in HAF are more likely to report beneficial outcomes than those with children not enrolled in HAF. In addition to examining the potential benefits of HAF participation we also determine whether parents and caregivers whose children spend more time in HAF are more likely to report more positive outcomes than parents and caregivers who children who spend less time in HAF.

## Background

In the UK during the year 2022 to 2023 official government statistics show that approximately 12 million people were in absolute poverty, equivalent to 18% of the UK population, including 3.6 million children. In addition, the number of households experiencing high levels of household food insecurity has worsened in recent years, with 3.7 million people reporting severe food insecurity in 2022–23, up by 1.5 million on the previous year (4). School lockdowns during the coronavirus pandemic also disrupted children’s education and social environments leading to, poor dietary habits, poor mental health and wellbeing and a reduction in physical activity (5–7). It is, therefore, hardly surprising that children and adults in low-income households are likely to have a poor diet and are more likely to be overweight or obese with associated poorer health outcomes both physically, mentally, and socially compared to their more affluent peers (8–12). The need for governments to address childhood poverty and associated high levels of household food insecurity and health inequalities has never been greater.

In the UK, several government-led initiatives have been implemented across the school day, including free school meals (FSM), universal infant free school meals (UIFSM) and school breakfast clubs (13). These programs aim to improve children’s health, wellbeing and educational attainment. FSM provide a nutritional safety net for many families during school term-time, with 23.8% of school children across England (2 M) eligible for FSM or Universal Infant Free School Meals in the 2022/2023 academic year (1) and there is emerging evidence that FSM may also mitigate household food insecurity by alleviating financial strain on households (14). However, the FSM and the UIFSM programs are not available during the school holidays, increasing the probability that children’s dietary intake will become poorer across the school holidays (15).

An additional challenge for families during the school holidays is finding affordable childcare. While the Childcare Act 2006 requires local authorities in England and Wales to ensure sufficient childcare is available for parents with children up to the age of 14 years, a number of surveys illustrate a lack of affordable childcare, particularly across the school holidays (16, 17). Moreover, an investigation by a joint Department for Work and Pensions and Education Select Committee of the House of Commons into poverty during the school summer holiday period heard evidence from parents who said that the requirement to pay child care costs up front and then claim them back through Universal Credit prevented them from being able to work during the summer holiday period and the absence of FSM meant they relied on food aid from food banks to feed their children during the summer holidays (10).

In response to the needs of underserved families during school holidays, many charities and community organizations established holiday clubs that were free for children to attend. These clubs alleviated the financial strains on household budgets (12, 18–21); reduced the risk of families experiencing household food insecurity (22, 23); improved children’s dietary intake (15, 24); increased physical activity (25, 26), provided nutritional education (3); and helped families with childcare provision (27). In addition, most holiday clubs offer numerous additional resources that improve the wellbeing for parents, children, volunteers, and staff including reducing parental stress, reducing parent and child social isolation and providing a safe place to play (27, 28).

However, such provision tended to be piecemeal (17), and it was not until 2021, following the success of a series of pilot programs (29, 30) that the DfE announced funding of £220 M p.a. for a Holiday Activities and Food (HAF) program across all 152 upper-tier local authorities in England (13). The DfE publish guidance to local authorities and holiday clubs regarding what HAF should cover. Specifically, local clubs must provide children with access to 60 min of physical activity, fun activities, and free, nutritious food each day. Families can also benefit through participating in cookery classes, social activities, take home food boxes, signposting toward other sources of information, and free childcare.

## Previous research on HAF

The national evaluations of HAF, commissioned by the DfE, show that approximately 50,000 children attended HAF in 2018, and 750,000 children in 2021, with 41 to 61% eligible for FSM. Survey data showed that parents and children thought HAF improved children’s skills, knowledge, socialization and wellbeing, and provided financial relief to households (29, 30). However, there are a number of limitations to these government funded reports. For example, management data sent to the DfE from each participating LA showed that around 750,000 children attended HAF in 2021, with most of primary school age (76%). A national HAF report by Cox et al. (30), experienced difficulties in recruiting a representative sample of HAF attendees as only those receiving free school meals were included in the sample. Moreover, fewer than 559 questionnaires in 16 local authorities were completed by parents and carers of parents and caregivers of HAF attendees, meaning there was an average of only 35 parents and caregivers sampled in each local authority. While a total of 4,437 surveys were completed by the comparator group of parents and caregivers (whose children did not attend HAF) data was not collected about important issues such as parental wellbeing, household food insecurity or physical activity according to levels of attendance. HAF attendees were also not.

At the local level, specific regions have also undertaken independent HAF evaluations (26, 31–33). However, most of these evaluations do not include a comparator group nor have any research programs researched whether the number of days that a child attends HAF is associated with changes in outcome measures. This paper aims to (a) explore whether there are differences in outcome measures according to group (Attending HAF vs. Non-Attendees), (b) examine potential differences in outcome measures according to level of HAF attendance.

## Data and methods

### Research design

Research examining the potential benefits of public services for vulnerable populations is notoriously challenging due to difficulties in accessing target populations and identifying an appropriate control group—i.e., participants who are eligible for but do not use the service (34).

**Figure 1:**
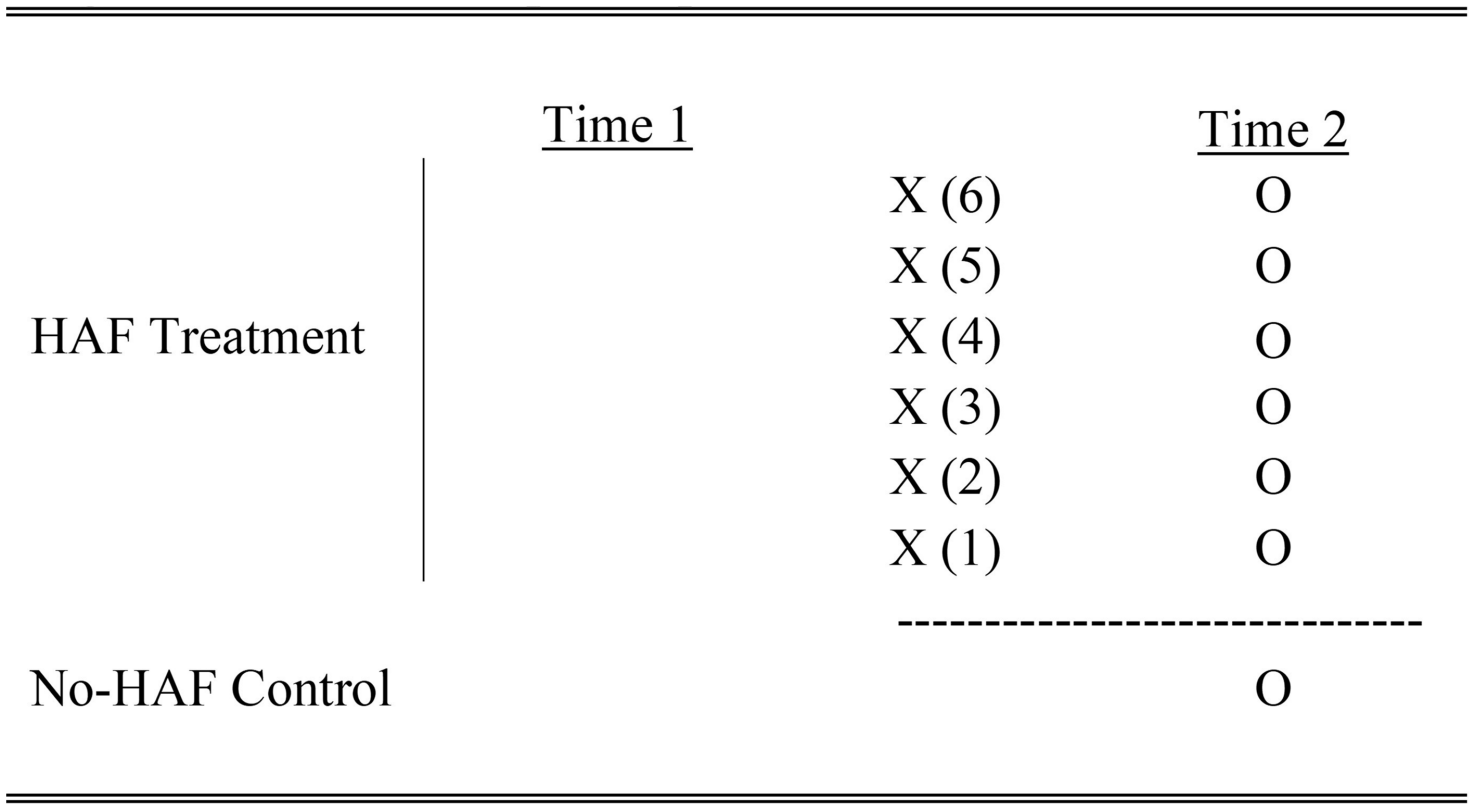
Static-group comparison HAF vs. No. HAF.

It is within this complex HAF milieu that we undertake our evaluation of potential HAF outcomes while attempting to minimize the impact of our research on resource strained HAF service providers. To do this we examine pre-existing HAF groups exposed to different amounts of HAF provision and compare them to a control group after the fact. This non-random static-group comparison is a type of quasi experimental design often used by researchers undertaking different types of evaluation (35–37). In the present study we model outcomes for six HAF groups that we believe produce different experiences according to the amount of time young people spend in HAF. This design is depicted in Figure 1.

As Figure 1 shows, there are no observations (i.e., “O”) taken at “Time 1” for any of the treatment groups X (1) - X (6) or the control group. The practical advantage of this design is that it minimizes the impact our research had on HAF service providers who struggle to collect data at the beginning of their HAF offering. Instead, observations are collected at Time 2, where each observation for the treatment group is compared to the observation for the control group. Given that we are undertaking 6 comparisons we apply a Bonferroni Correction when conducting our statistical analysis according to the following formula: *α* = 0.05/6.

This quasi-experimental design and does have limitations. Most importantly, because randomization is not used to assign membership to the control or treatment groups there may be differences in those groups. Nevertheless, static group designs are do have advantages as they are relatively unobtrusive, by using only one data collection period. This was a priority in our research as we wanted to minimize the impact of our research on HAF providers. Second, the use of treatment and control groups in a static group design allows us to safely assume that any differences between HAF groups and our control group are probably not due to testing, regression to the mean, history or survey instrumentation (37). Third, our research instrument (described below) allowed us to collect additional information to adjust our estimates for potential treatment and control group differences. Thus, we can also be relatively certain, when these adjustments are applied, that group differences are not driving our findings. Nevertheless, the lack of equivalent groups through randomization means we need to be cautious when drawing specific causal inferences.

### Sample

We sampled (1) parents and caregivers of children who attended HAF (Attending HAF) and (2) parents and caregivers of children who were eligible, but did not, attend HAF (Non-Attendees). Questionnaires were distributed to both groups of parents/caregivers at the end of the summer holidays in September of 2021 and September of 2022 so that participants could reflect on their experiences of food, wellbeing, physical activity, safety, and childcare over the summer. While our sampling methods did not allow us to follow the same parents/caregivers across two years of data collection, we were able to estimate the potential changes in the impact of HAF between 2021 and 2022. Collecting data for two years was important given the global pandemic that produced a quickly changing service environment that might have altered the behavior of parents, caregivers, children and HAF providers between 2021 and 2022. Thus, adjusting for time as a potential confounder allowed us to control for this potentially important non-program related difference.

As noted, our sample consists of parents/caregivers with at least one child attending HAF during the 2021 and 2022 school summer holidays as well as parents and caregivers whose children are not attending HAF. We recruited parents and caregivers whose children attended HAF by working with a HAF coordinator who had responsibility of HAF at the local authority level. The coordinator helped us with participant recruitment by asking 20 HAF clubs to send a recruitment e-mail (for those with an e-mail on record) to parents and caregivers of children that attended at least one HAF session (*N* ≈ 2000 potential respondents each year). That email was sent in September 2021 and again in September 2022 and contained an embedded hyperlink leading them to a short questionnaire about HAF attendance as well as perceptions about food insecurity, wellbeing, physical activity, safety and childcare and individual/household demographics.

We also surveyed the parents and caregivers of non-Attendees. These links were also distributed via email using the online survey platform Prolific^1^ in September 2021 and again in September 2022. To ensure parents and caregivers of non-Attendees, were eligible but not registered to attend HAF, we asked Prolific to produce a representative group of parents and caregivers of children aged 4 to 16 whose annual income was less than or equal to £19,999 before housing costs and who had not registered for HAF (around *N* ≈ 1,750 potential respondents each year). This income cutoff for households with children generally corresponds to the cutoff for HAF attendance. Prolific emailed these parents and caregivers a link to participate in the survey. Participants decided if they wanted to participate in our research on a first come first served basis.

Overall, the study sample composed of *n* = 1,621 parents and caregivers. Of those participants who completed the questionnaire, 736 had children attending HAF (response rate of approximately 18.4%) and 885 had child/children not attending HAF (response rate of approximately 38.2%).

### Variables in analysis

An online questionnaire was used to collect data for the variables in this analysis. Ethical approval to undertake this study was granted by Northumbria University (Number 33684). All participants were informed that their participation in the research was voluntary and that if they did participate that they were under no obligation to answer all the survey questions. Below we describe the variables used in our statistical analysis.

#### Outcome variables

The present examination looks at five outcomes that parents and caregivers say are the benefits of HAF (28). These potential HAF benefits are (1) greater levels of physical activity, (2) increased household food security, (3) more affordable childcare, (4) better parent wellbeing and (5) increased perceptions of safety for children while they play. To evaluate these potential benefits, we create five outcome variables. Descriptive statistics (mean, median, range and standard deviation) for these variables (and all other variables in this research) are included in Appendix A.

*Physical Activity* is created by asking parents and caregivers to estimate the number of weeks during the summer that their child exercised *“at least four times a week for at least 60 min during the day*.” Parent and caregiver estimates ranged from ‘0’ weeks of exercise of exercise at least four days a week at least 60 min a day to ‘6’ weeks of exercise of at least four days a week at least 60 min a day. In general, higher scores for the variable physical activity are more beneficial for children’s health.

The second outcome variable we created is *Household Food Security*. This variable is derived using an adaptation to the 6-item US Household Food Security Survey Module (38). However, we alter that measure of food security to estimate food insecurity over the summer school holiday, which is approximately 6 weeks (as opposed to the USDA measure which looks at the previous 12 months or previous 30 days). Participants were asked how often during the school holidays (1) “*The food we bought just did not last, and we did not have money to get more*” and (2) how often “*We could not afford to eat balanced meals*.” Participants were also asked if they (3) “*Cut the size of their meals or skipped meals because there wasn’t enough money for food*” and (4) if they “*Eaten less than they felt they should because there wasn’t enough money for food*.” Those participants who responded “yes” to the last two questions were then asked how frequently these situations occurred during the school summer holidays. The USDA’s measure of food insecurity is the one most often used measure of household food security and researchers have validated it in multiple countries (39). *Household Food Security* is an additive outcome representing a count of the number of affirmative answers to the six food insecurity items such that higher scores represent higher levels of food insecurity. In the present study we reverse code this scoring system to be consistent with other outcomes. Thus, higher scores are more desirable and indicate *more* food security with a score of ‘0’ suggesting very low food security and a score of ‘6’ indicating high levels of food security.

*Affordable Childcare* is the third outcome measure we examine. To measure this variable, we ask participants, “*How difficult was it to find affordable childcare over the most recent summer school holiday*?” Participants estimated childcare difficulty on a scale ranging from ‘0’ (very difficult) to ‘10’ (not difficult).

The fourth outcome measure is *Parent Wellbeing* and is based on the ‘Perceived Stress Scale’ Cohen et al. (40). Participants were asked how often during the summer that they felt they were (1) *unable to control the important things in their life*; (2) *felt confident in their ability to handle personal problems*; (3) *felt things were going their way* and (4) *felt difficulties were piling up so high that they could not be overcome*. Answers to each item ranged from ‘Never’ to ‘Almost Always’ and were scored on a scale of 0 to 4. We added the scores on these items together after reverse coding scores for items 1 and 4. Thus, high scores reflected greater wellbeing. That is, *Parent Wellbeing* scores ranged from 0 (extreme levels of stress) to 16 (no stress). The Perceived Stress Scale has been validated and used in a wide range of countries and settings (e.g., (41, 42)).

The fifth outcome measures perceptions of safety. We ask whether children have a *Safe Place to Play* during the summer school holiday. To measure this variable, we ask parents and caregivers, “*How safe do you feel your children are in your neighbourhood during the summer*?” Respondents answered using a scale ranging from ‘1’ (not safe) to ‘5’ (very safe).

#### Time spent in HAF

This study includes one main predictor variable to test the impact of time spent in HAF. This predictor variable is made up of 7 categories that represent 7 groups (i.e., 1 control group and 6 treatment groups). The first group is the Non-Attendee group (i.e., the control group) and consists of children who did not attend HAF. The remaining 6 categories represent the 6 different HAF treatment groups, each of which can be compared to the Non-Attendee group. To create these 6 treatment groups, we first calculated the total number of hours each child spent in HAF. Parents and caregivers were asked to estimate, on average, how many weeks in the summer, how many days per week, and how many hours per day their children attended HAF. This information allowed us to estimate the number of hours each child spent in HAF so that we could reliably organize children into treatment groups. To do this we draw upon the 4×4 service model (43). In short, 16 h of attendance (i.e., 4 h a day for 4 days per week) is equivalent to attending 1 week of HAF. Based on this 4×4 model we categorized the 6 HAF treatment groups as follows: (1 Week) 1 to 16 h (*n* = 79); (2 Weeks) 17–32 h (*n* = 101); (3 Weeks) 33–48 h (*n* = 118); (4 Weeks) 49–64 h (*n* = 135); (5 Weeks) 65–80 h (*n* = 87); (6 Weeks) 80–240 h (*n* = 123).

#### Control variables

To better assess potential correlation between *Time Spent in HAF* and the outcome variables we control adjust our estimates for potential group differences. To do this we include a set control variable that distinguish between the treatment and control group. These control variables include (1) household characteristics, (2) respondent demographics and (3) a time indicator representing the summer studied to account for trends over time. First, we measure economic deprivation using a variable labeled *Free School Meal Eligible.* This is a dichotomous indicator scored 1 = ‘yes’ and 0 = ‘no’ where an affirmative response indicated that the household income was low enough to qualify for free school meals. Parents/caregivers who are economically disadvantaged are (1) more likely to suffer from lower levels of food security, (2) more likely to have a hard time finding affordable childcare, are (3) more likely to face parental stress and are (4) more likely to live in areas where they may perceive that their children are not safe when unsupervised. HAF targets households where income levels are low enough for any children in the household to qualify for means tested free school meals. Second, we control household financial strain by identifying households where the primary income earner is out of work (i.e., *Primary Earner Unemployed & Seeking Work*). This variable is also dichotomous and scored ‘1’ when the primary household earner is classified as unemployed and seeking work and ‘0’ if they are not seeking work because they are employed, retired or receiving benefits. Households where primary earners are unemployed are less likely to be food secure, and parents in those households are more likely to suffer from parental stress (12). At the same time, households where the primary wage earner is unemployed may be more likely to attend HAF because of their financial situation. Third, *Single Parent Households* may have a more difficult time affording food and childcare, while parents may have less time to ensure their children get enough exercise and may face higher levels of stress (9). *Single Parent Households* is coded as ‘1’ when the household identifies as a single parent and ‘0’ when the household does not identify as a single parent. Again, single parent households may be more likely to send children to HAF if given the chance but have lower levels of food security and higher levels of stress. Fourth, the *Household Size* is simply an indicator of the number of people living in the household. As the number of people in a household increases there may be more pressure on the household for resources such as food, money for childcare and potentially more pressure on parents to provide these things (9, 44).

Respondent demographics variables included *gender*, *age* and *ethnicity.* To measure *gender*, participants were asked to report their gender measured as ‘female’, ‘male’, ‘non- binary’, ‘third gender’ or ‘self-describe’. Given the relatively low frequencies for individuals who were non-binary, third gender or self-describe we decided to code gender using a dummy variable where ‘1’ indicates the respondent identifies as male while ‘0’ indicates the respondent does not identify as male (i.e., ‘female’, ‘non-binary/third gender’ or ‘self- described’ their gender). *Age* is measured in years. Finally, *race/ethnicity* is measured by employing the UK’s official ethnic categories (i.e., white [English/Welsch/Scottish/Northern Irish/British, Irish, Other White Background], Asian [Indian, Pakistani, Bangladeshi, Chinese, other Asian background], and black [African, Caribbean, Any other black background], mixed/multiple ethnicities [white and black Caribbean, white and black African, white and Asian, any other mixed/multiple ethnicities], and any other ethnic group [Arab and any other ethnic group]). We report the proportion white in descriptive tables but treat ethnicity as a dummy variable in the multivariate analysis to estimate race/ethnicity and the effect of being (1) Asian, (2) Black, (3) Mixed/Multiple Ethnicity and (4) Other Ethnicity in comparison to (5) Whites (the omitted category). We also created a control variable that serves as an indicator for the year in which the questionnaire was distributed. This variable accounts for changes between 2021 and 2022 during the Covid pandemic that may have influenced *Physical Activity*, *Household Food Security*, *Affordable Childcare*, *Parent Wellbeing* and *Perceived Safety* (45–47).

### Analytic strategy

We use ordinary least squares regression (OLS) to evaluate the potential impact of *Time Spent in HAF* (i.e., comparing the different HAF treatment groups to the non-HAF control group) for the five outcome variables: (1) *Physical Activity*, (2) *Household Food Security*, (3) *Affordable Childcare*, (4) *Parent Wellbeing* and (5) *Perceptions of Safety*. However, OLS, while robust, is appropriate for continuous outcome variables. In the present analysis some of the outcome variables are ordinal in nature (i.e., affordable childcare and perceptions of safety). Other outcome variables such as *Household Food Security* and *Parental Wellbeing* are often created from multiple items and therefore often treated as continuous by researchers (12). As OLS might produce misleading results, we replicate that analysis using Ordinal Logistic Regression [OLR; see (48)]. However, OLR results are substantively the same and included in Appendix B for readers who prefer this approach.

Our strategy is to examine each outcome in a separate analysis and present the results in series of 5 tables organized by outcome. The first step is to only model *Time Spent in HAF* on the outcome. Second, we evaluate *Time Spent in HAF* on that outcome while adjusting for differences between the HAF treatment group and the non-HAF control group. We then compare coefficients for *Time Spent in HAF* generated in step one to coefficients for *Time Spent in HAF* generated in step two. This comparison gives us some indications about the impact of controls on the analysis (with small changes in the coefficients indicating more confidence in the results). That is, by adjusting for group differences we can explore whether non-equivalent treatment and control groups could be the reason for any observed association between *Time Spent in HAF* and *Physical Activity*, *Household Food Security*, *Affordable Childcare*, *Parent Wellbeing* and/or *Perceptions of Safety*. The statistical software *Stata V15* was used for all statistical analysis. Each table includes the unstandardized regression coefficient (for ease of interpretation) for each variable in the model along with the 95% confidence intervals around those coefficients to estimate the largest potential impact any treatment could have. In the case of *Time Spent in HAF*, these coefficients can be interpreted as the unit change in the outcome across the sample when a parent or caregiver’s child attends a HAF treatment group as opposed to being part of the non-HAF control group. Finally, to aid in interpretability we also include Appendix C, which includes means for the non-HAF control group and HAF treatment groups.

As we are comparing multiple groups, we apply a Bonferroni Correction to reduce Type 1 error prior to examining the potential impact of *Time Spent in HAF* on outcomes.

When estimating each model, we also checked regression assumptions to ensure they are satisfied. We find that the error term in each model appears to be normally distributed and homoscedastic and there is little evidence of multicollinearity in any model estimated. We also tested for interactions between *Time Spent in HAF* and each control variable (analysis not shown) but found little evidence that any interactions existed in these data.

## Results

The results of our analysis are presented in five different tables—one table for each outcome. The outcomes we investigate are (1) *Physical Activity*, (2) *Household Food Security*, (3) *Affordable Childcare,* (4) *Parent Wellbeing*, and (5) *Perceptions of Safety*. Prior to presenting the results of those five analyses we compare basic demographic characteristics for the local authority, the Attending HAF treatment group and the Non-Attending control group (see Table 1). Table 1 compares the basic demographic characteristics of the local authority, the Attending HAF treatment group, and the Non-Attending control group. Statistical significance tests for differences in means (*t*-tests) and proportions (z-tests) between the treatment and control groups are included in the last column of Table 1.

**Table 1:** Comparing demographic variables in the population, HAF and non-HAF groups, 2021/2022.

	Local (a, b)	Attending HAF	Non-Attendee	Test statistic for differences in independent samples: Treatment vs. Control (c)
	Authority (Population)	Group (Treatment) (*n* = 735)	Group (Control) (*n* = 885)	
Household characteristics				
Free school meals eligible	25.8%	76.1%	57.5%	z = 7.86*
Primary earner unemployed	7.2%	14.0%	6.3%	z = 5.37*
Single parent household	15.2%	36.0%	44.3%	z = −3.70*
Household size	2.4	4.3	3.6	t = 10.3*
Survey respondent characteristics				
Gender (Female)	51.6%	88.3%	82.1%	z = −6.38*
Mean Age (in years)	34.0	38.9	36.2	t = 1.81
Race/Ethnicity				
White	53.3%	43.1%	82.4%	z = −20.1*
Asian, Asian British or Asian Welsh	28.8%	32.1%	6.9%	z = 14.0*
Black, Black British	–	–	–	–
Black Welsh, Caribbean or African	10.0%	10.5%	5.3%	z = 4.17*
Other Ethnic Groups	4.6%	5.9%	1.9%	z = 4.36*
Mixed or Multiple ethnic groups	3.3%	8.4%	3.5%	z = 3.95*

(a) Department for Education (57). (b) Office of National Statistics (58). (c) *p < 0.05.

Table 1 contains evidence that the Attending HAF group and Non-Attendees group are, as expected, more economically marginalized than the general population in the local authority. For instance, while 25.8% pupils in the local authority are eligible for FSM, 76.1% of those in the Attending HAF group and 57.5% of those in the Non-Attendee group are eligible for FSM. This is expected as FSM is often a criterion used to screen HAF enrolment. Thus, compared to households in the general population, both the Attending HAF group and the Non-Attendee group are more likely to be economically marginalized given the distribution of FSM across the population and sample. While we attempted to obtain a Non-Attendee group that is equivalent to the Attending HAF group, these groups are not, however, as equivalent as we might like. In particular, the Attending HAF group has a greater percentage of free school meal youth than the Non-Attendee group (i.e., 18.6% or 76.1–57.5%). Thus, it is important to control for FSM in any HAF analysis of outcomes. Moreover, Attending HAF and Non-Attendee groups have larger percentages of single parent households (i.e., 36.0 and 44.3%) than the population (15.2%). Again, this provides evidence that the treatment and control groups probably face more parenting challenges, on average, than the local authority population. Nevertheless, the Non-Attendee group has a greater percentage of single parent households than the Attending HAF group. While unemployment among parents and caregivers is higher in the Attending HAF group (i.e., 14.0%) it is closer to equivalent in the population than the Non-Attendee group (7.2% vs. 6.3%), meaning the Non-Attendee group may be composed of more working poor than the general population. Finally, and as expected, household size is larger among the Attending HAF group (mean = 4.3 people) and the Non-Attending control group (mean = 3.6 people) than the overall local authority population (mean = 2.4 people) given the presence of children and young people in the sample. There are also differences in racial/ethnic demographics among the population and the two groups. In the local authority 53.3% of the population is White compared to 43% of the Attending-HAF parents and caregivers and 83% of the Non-Attendee parents and caregivers reporting they were White. Finally, it is important to note that the differences in means and proportions between the Attending-HAF and Non-Attendee groups are statistically significant at *p* < 0.05 for all demographic variables in our analysis except *Age*, meaning that the two samples do not come from the same population of parents and caregivers. As a result, these group differences must be accounted for in any comparisons between the level of HAF activity and potential HAF outcomes.

### Physical activity

In terms of outcome measures, we examined the association between *Time Spent in HAF* and *Physical Activity*. Table 2 presents the OLS regression results examining the relationship between *Time Spent in HAF* and parent/caregiver estimates of the number of weeks children engage in physical activity during the summer. According to the coefficients in Model 1 (Table 2), parents of children who attend HAF for 1 week (i.e., the ‘1 Week’ HAF treatment group) report no significant difference in physical activity levels compared to parents of children in the non-HAF control group. A similar pattern is observed for children who attend HAF for 2 weeks and 3 weeks. Furthermore, even after adjusting for control variables in Model 2 (Table 2), we find no evidence to suggest that children who attend HAF for up to 3 weeks are more physically active than non-attendees.

**Table 2:** Ordinary least squares regression exploring the relationship between HAF attendance (2021–2022) and parent perceptions of physical activity in a UK local authority.

	Model 1	Model 2
	Parent estimate of weeks of physical activity	Parent estimate of weeks of physical activity
	95% CI	95% CI
	b (LL, UL)	b (LL, UL)
Time spent in HAF (vs. didn't attend)		
1 Week or Less	−0.26 (−0.69, 0.18)	−0.18 (−0.66, 0.31)
2 Weeks	−0.14 (−0.53, 0.26)	0.12 (−0.31, 0.54)
3 Weeks	−0.10 (−0.47, 0.27)	0.29 (−0.12, 0.69)
4 Weeks	0.59 (0.25, 0.94)*	1.00 (0.61, 1.39)*
5 Weeks	0.76 (0.33, 1.18)*	1.15 (0.69, 1.62)*
6 Weeks or More	1.48 (1.12, 1.84)*	1.97 (1.57, 2.36)*
Household characteristics		
Free school meals eligible		0.02 (0.20, 0.24)
Primary earner unemployed		0.18 (−0.16, 0.52)
Single parent household		0.21 (0.00, 0.43)
Household size		−0.05 (−0.13, 0.03)
Respondent characteristics		
Gender (1=Male)		−0.18 (−0.45, 0.10)
Age (in years)		−0.02 (−0.03, 0.00)
Race/Ethnicity (vs. White)		
Asian		−0.89 (−1.18, −0.59)
Black		−0.67 (−1.05, −0.28)
Other ethnic group		−0.82 (−1.37, −0.26)
Mixed ethnicity		−0.75 (−1.20, −0.31)
Year (vs. 2020)		
2021	0.50 (−0.13, 0.26)	0.03 (−0.17, 0.23)
Constant	2.74 (2.58, 2.90)	3.63 (2.98, 4.28)
Adj. R-Square	0.05	0.09
Sample size (*n*)	1,615	1,453

Bolded (*) results for time spent in HAF are “statistically significant” under the Bonferroni Correction or *p* < 0.008 (i.e., α = 0.05/6).

However, this trend changes for children who participate for more than three weeks. For example, according to the coefficients in Model 1 (Table 2), parents of children who attend HAF for 4 weeks (i.e., the ‘4-Week’ HAF treatment group) report that their children engage in 0.59, 95% CI [0.25, 0.94] more weeks of physical activity compared to children in the Non-Attendee group. This coefficient is larger for 5 weeks (*b* = 0.76, 95% CI [0.33, 1.18]) and larger still for 6 weeks (*b* = 1.48, 95% CI [1.12, 1.84]). Moreover, when adjusting for group differences in Model 2 (Table 2) the potential impact of *Time Spent in HAF* intensifies. For instance, according to results in Model 2 parents and caregivers estimate that children who attend HAF 6 weeks report their children engage in 1.97, 95% CI [1.57, 2.36] more weeks of physical activity compared to children in the Non-Attendee group. In short, the benefits of HAF on increased physical activity emerge when children attend HAF for 16 h per week for at least 4 weeks. It is also notable that Table 2 (Model 2) suggests that Asian, Black, mixed or other ethnic groups engage in 0.67 to 0.87 fewer weeks of physical activity than Whites. This finding is not unusual, but consistent with past literature on Black and minority ethnic groups in the United Kingdom (49).

### Household food security

The coefficients summarizing the relationship between *Time Spent in HAF* and parent/caregiver estimates of household food insecurity are in Table 3. Parents of children who attend HAF report no significant difference in household food insecurity compared to parents of children in the non-HAF control group. This finding is consistent across each HAF treatment group and consistent across Models 1 and 2. Thus, we find no evidence that children who attend HAF are more likely to live in food insecure houses than children who do not attend HAF.

**Table 3:** Ordinary least squares regression exploring the relationship between HAF attendance (2021–2022) and household food insecurity in a UK local authority.

	Model 1	Model 2
	Household food security	Household food security
	95% CI	95% CI
	b (LL, UL)	b (LL, UL)
Time spent in HAF (vs. didn't attend)		
1 Week or Less	0.50 (−0.02,1.02)	0.33 (−0.25, 0.90)
2 Weeks	0.22 (−0.25, 0.69)	0.07 (−0.44, 0.58)
3 Weeks	−0.04 (−0.48, 0.40)	0.04 (−0.44, 0.52)
4 Weeks	−0.38 (−0.79, 0.03)	−0.54 (−1.00, −0.07)
5 Weeks	0.01 (−0.49, 0.52)	0.06 (−0.49, 0.62)
6 Weeks or More	−0.27 (−0.70, 0.16)	−0.31 (−0.78, 0.16)
Household characteristics		
Free school meals eligible		−0.96 (−1.22, −0.70)
Primary earner unemployed		−0.29 (−0.70, 0.11)
Single parent household		−0.24 (−0.50, 0.01)
Household size		−0.07 (−0.16, 0.02)
Respondent characteristics		
Gender (1=Male)		−0.14 (−0.47,0.20)
Age (in years)		0.02 (0.01,0.04)
Race/Ethnicity (vs. White)		
Asian		0.48 (0.13, 0.83)
Black		0.24 (−0.22, 0.70)
Other ethnic group		1.14 (0.48, 1.81)
Mixed ethnicity		0.05 (−0.48, 0.58)
Year (vs. 2020)		
2021	−0.914 (−1.14, −0.69)	−0.86 (−1.10, −0.63)
Constant	3.986 (3.79, 4.18)	3.99 (3.21, 4.76)
Adj. R-Square	0.04	0.09
Sample size (*n*)	1,618	1,455

Bolded (*) results for time spent in HAF are "statistically significant" under the Bonferroni Correction or *p* < 0.008 (i.e., α = 0.05/6).

Four control variables in Model 2 (Table 3) are correlated with food insecurity. First, and as might be expected, youth who receive *Free School Meals* score 0.96 points, 95% CI [−1.22, −0.70] lower on the *Household Food Security* index. This result indicates that pupils who are eligible for free school meals tend to live in less food secure households. Second, older survey respondents tend to live in slightly more food secure households (i.e., 0.02, 95% CI [0.01, 0.04]). For instance, across the sample a one standard deviation increase (i.e., 7 years) in *Age* is associated with a 0.14-point increase in *Household Food Insecurity*. Finally, survey respondents who are *Asian* or identify as belonging to an *Other Ethnic Group* tend to be more food secure than respondents who identify as *White*.

### Affordable childcare

Table 4 lists the regression coefficients examining the relationship between *Time Spent in HAF* and perceptions of *Affordable Childcare*. According to the coefficients in Model 1 (Table 4), parents/carers of children who attend HAF for 1 to 5 weeks report no significant difference in perceptions about Affordable Childcare compared to parents/carers in the Non-Attendee group.

**Table 4:** Ordinary least squares regression exploring the relationship between HAF attendance (2021–2022) and perceptions of affordable childcare in a UK local authority.

	Model 1	Model 2
	Affordable Childcare	Affordable Childcare
	95% CI	95% CI
	b (LL, UL)	b (LL, UL)
Time spent in HAF (vs. didn't attend)		
1 Week or Less	0.40 (−0.51,1.31)	0.18 (−0.83,1.19)
2 Weeks	0.04 (−0.82,0.90)	−0.23 (−1.19, 0.73)
3 Weeks	0.19 (−0.57, 0.96)	0.14 (−0.73, 1.00)
4 Weeks	−0.52 (−1.26, 0.21)	−0.68 (−1.52, 0.15)
5 Weeks	−0.06 (−0.91, 0.80)	−0.28 (−1.25, 0.69)
6 Weeks or more	−1.33 (−2.07, −0.59)*	−1.35 (−2.18, −0.51)*
Household characteristics		
Free school meals eligible		−0.16 (−0.66, 0.34)
Primary earner unemployed		−0.64 (−1.40, 0.13)
Single parent household		0.01 (−0.48, 0.49)
Household size		−0.06 (−0.23, 0.11)
Respondent characteristics		
Gender (1=Male)		−0.81 (−1.44, −0.19)
Age (in years)		0.02 (−0.01, 0.06)
Race/Ethnicity (vs. White)		
Asian		0.56 (−0.07, 1.19)
Black		−0.10 (−0.93, 0.74)
Other ethnic group		−0.12 (−1.36, 1.13)
Mixed ethnicity		0.75 (−0.25, 1.75)
Year (vs. 2020)		
2021	−0.11 (−0.53,0.32)	−0.10 (−0.56, 0.35)
Constant	6.57 (6.20, 6.94)	6.17 (4.67, 7.68)
Adj. R-Square	0.01	0.03
Sample size (*n*)	1,618	1,008

Bolded (*) results for time spent in HAF are "statistically significant" under the Bonferroni Correction or *p* < 0.008 (i.e., α = 0.05/6).

However, there is a significant association between the 6 Week HAF treatment group and the Non-Attendee group. That is, coefficients in Model 1 (Table 2) suggest that parents of children who attend HAF for 6 weeks are more concerned about affordable childcare than parents of children in the Non-Attendee group −1.33, 95% CI [−2.07, −0.59]. This finding is consistent with the finding in Model 2 that adjusts for controls (i.e., −1.35, 95% CI [−2.18, −0.51]). In short, across the sample of parents and caregivers those who attend HAF for six weeks (or longer) are, on average, 1.3 points *more* concerned about affordable childcare than parents and caregivers of children who did not attend HAF. This finding is unexpected given that that it is only those parents and caregivers that send their children to HAF for 6 weeks who are concerned about affordable childcare. As a result, we suggest that this finding needs additional exploration. It might be, for instance, that more concerns about the high costs of childcare are driving parents and caregivers to spend more time in HAF.

### Parent wellbeing

Table 5 examines the relationship between *Time Spent in HAF* and *Parental Wellbeing*. The coefficients in Model 1 (Table 4) suggest that parents and caregivers of children who attend HAF for 1 to 5 weeks report no significant difference in self-reported *Parental Wellbeing* compared to parents and caregivers in the Non-Attendee group.

**Table 5:** Ordinary least squares regression exploring the relationship between HAF attendance (2021–2022) and parental wellbeing in a UK local authority.

	Model 1	Model 2
	Parent wellbeing	Parent wellbeing
	95% CI	95% CI
	b (LL, UL)	b (LL, UL)
Time spent in HAF (vs. didn't attend)		
1 Week or Less	0.92 (0.24, 1.60)	0.81 (0.04, 1.58)
2 Weeks	0.79 (0.18,1.40)	0.57 (−0.12,1.25)
3 Weeks	0.53 (0.05,1.10)	0.54 (−0.10, 1.19)
4 Weeks	0.61 (0.07, 1.15)	0.51 (−0.12, 1.13)
5 Weeks	0.57 (−0.09,1.23)	0.57 (−0.17,1.32)
6 Weeks or More	1.12 (0.56,1.69)*	1.06 (0.42,1.70)*
Household characteristics		
Free school meals eligible		−0.73 (−1.08, −0.39)
Primary earner unemployed		−0.48 (−1.02, 0.07)
Single parent household		0.05 (−0.29, 0.39)
Household size		0.10 (−0.02,0.22)
Respondent characteristics		
Gender (1=Male)		−0.18 (−0.63, 0.27)
Age (in years)		0.01 (−0.01, 0.03)
Race/Ethnicity (vs. White)		
Asian		0.10 (−0.37, 0.57)
Black		0.71 (0.08, 1.33)
Other ethnic group		0.73 (−0.17, 1.63)
Mixed ethnicity		0.16 (−0.55,0.87)
Year (vs. 2020)		
2021	−0.64 (−0.94, −0.34)	−0.58 (−0.90, −0.26)
Constant	8.00 (6.20, 8.49)	7.89 (6.84, 8.93)
Adj. R-Square	0.02	0.01
Sample size (*n*)	1,615	1,453

Bolded (*) results for time spent in HAF are "statistically significant" under the Bonferroni Correction or *p* < 0.008 (i.e., α = 0.05/6).

However, there is a significant association between the 6 Week HAF treatment group and the Non-Attendee group. That is, coefficients in Model 1 (Table 2) suggest that parents and caregivers of children who attend HAF for 6 weeks or more score 1.12, 95% CI [0.6, 1.69] points more on the Perceived Stress Scale (higher scores, less stress) than parents of children who do not attend HAF. Even when adjusting for control variables this coefficient changes little (i.e., 1.06, 95% CI [0.42,1.17]).

Two control variables in Table 5 (Model 2) are also correlated with *Parental Wellbeing*. First, youth who receive *Free School Meals* score 0.73, 95% CI [−1.10, −0.39] points lower on the *Parental Wellbeing* variable. Across the sample, parents of young people eligible for free school meals tend to have lower levels of wellbeing. Second, parents who completed the survey in 2022 tended to have lower levels of wellbeing than parents who completed the survey in 2021 (−0.58, 95% /CI [−0.90, 0.26]). Returning from Covid lockdown may have proved to be an especially stressful time for parents and caregivers.

### Perceptions of safety

Table 6 examines the relationship between *Time Spent in HAF* and *Perceptions of Safety*. The coefficients in Model 1 (Table 4) suggest that the parents and caregivers of attendees in the HAF treatment groups are no more or less likely to believe that children are safe in their neighborhoods than in the non-HAF control group. In particular, the only variable that appears to be associated with *Perceptions of Safety* is gender. Male participants, as is often predicted by the literature on risk (i.e., (50)), are more likely to believe that children are safe in their neighborhoods than female participants (−0.58, 95% /CI [−0.90, 0.26]).

**Table 6:** Ordinary least squares regression exploring the relationship between HAF attendance (2021–2022) and parent perceptions of safe place to play in a UK local authority.

	Model 1	Model 2
	Parent perceptions of safety	Parent perceptions of safety
	95% CI	95% CI
	b (LL,UL)	b (LL, UL)
Time spent in HAF (vs. didn't attend)		
1 Week or Less	0.04 (−0.24, 0.32)	0.13 (−0.19, 0.44)
2 Weeks	−0.24 (−0.48, 0.01)	−0.12 (−0.40, 0.15)
3 Weeks	−0.11 (−0.34, 0.12)	−0.08 (−0.34, 0.18)
4 Weeks	−0.16 (−0.38, 0.06)	−0.08 (−0.33, 0.17)
5 Weeks	0.09 (−0.18, 0.35)	0.18 (−0.12, 0.48)
6 Weeks or More	0.12 (−0.11, 0.34)	0.10 (−0.16, 0.35)
Household characteristics		
Free school meals eligible		−0.08 (−0.22, 0.06)
Primary earner unemployed		−0.03 (−0.25, 0.19)
Single parent household		0.05 (−0.08, 0.19)
Household size		0.00 (−0.05,0.05)
Respondent characteristics		
Gender (1=Male)		0.28 (0.10, 0.45)
Age (in years)		0.00 (−0.01,0.01)
Race/Ethnicity (vs. White)		
Asian		0.05 (−0.14,0.24)
Black		0.00 (−0.25,0.25)
Other ethnic group		−0.11 (−0.48,0.25)
Mixed ethnicity		−0.09 (−0.37, 0.20)
Year (vs. 2020)		
2021	0.02 (−0.10,0.14)	0.03 (−0.90,−0.26)
Constant	3.44 (3.34,3.54)	3.52 (−0.10, 0.15)
Adj. R-Square	0.01	0.01
Sample size (*n*)	1,585	1,423

Bolded (*) results for time spent in HAF are “statistically significant” under the Bonferroni Correction or *p* < 0.008 (i.e., α = 0.05/6).

## Discussion and conclusion

The overall findings of this study are mixed, and some are counter to those in prior research. While the parents/caregivers of HAF attendees report better parental wellbeing scores when their children attend HAF for six weeks or more, and increased participation in physical activity for their children when their children attend four weeks or more, compared to the comparator group, there were no observed differences between groups in terms of household food insecurity, and safe places for their children to play regardless of the time children spent at HAF. Furthermore, parents/caregivers whose children attend HAF for six weeks or more were more concerned about affordable childcare compared to non-attendees.

Some of these findings contradict prior findings that report HAF alleviates household food insecurity (10, 23), helps parents find affordable childcare (30) and provides safe places for their children to play (27).

A few early studies reported that HAF attendance alleviates household food insecurity (e.g., Holley et al., (10, 23, 31)), and a number of local authorities view HAF as a program that can be used to alleviate household food insecurity across the school holidays. However, all the prior studies were either pilot studies or relatively small in scale, and none employed a comparator group. It may be the case, as reported here, that HAF attendance does not attenuate household food insecurity, but it may also be the case that the HAF group in this study are deprived along multiple dimensions (e.g., FSM status) while the comparator group were selected purely on the basis of low household income. Thus, it may be the case that the HAF group may experience additional disadvantages, compared to the control group, not captured in this study, making it likely that the HAF group would be worse off if their children did not attend HAF. Without collecting pre- and post-HAF data, with a control group, these two proposals cannot be tested. It is important to note that, in the current study, the number of times a child attends HAF does not correlate with household food insecurity, so there is no evidence in our findings that increased HAF attendance is associated with lower household food insecurity. However, other research studies have shown that HAF provides an effective, nutritional safety net for children and young people across the school holidays (15, 24) and as such, it meets the DfE’s objective in terms of HAF providing children with access to nutritious food during the school holidays. Although see Campbell-Jack et al.’s (29) DfE report that found a decrease in healthy food consumption for HAF attendees versus non-attendees. This may be explained by the fact that Campbell-Jack et al. (29) collected parent’s self-reported perceptions regarding whether they thought the food was healthy, while the study by Crilley et al. (24) and Vitale et al. (15) conducted nutritional analysis on the actual food served and consumed.

In terms of childcare, our findings do not support the findings of a national HAF evaluation, commissioned by the DfE, that reports that parents whose child/children attend HAF find it easier to find affordable childcare compared to a comparator group of non-HAF attendees (29). Rather, we find that parents, on average, struggle to find affordable childcare whether their children attended HAF or not and that parents/caregivers whose children attend 6 weeks of HAF are significantly more concerned about finding affordable childcare than parents/caregivers of non-attendees. The reasons for the different research findings are unclear but may be driven by the smaller HAF sample in the national evaluation compared to the HAF sample in the current study. Given the overall increased costs for childcare provision across England, and the shortage of childcare providers, it may also be the case that parents/caregivers whose children attend HAF for six weeks simply have more general concerns about finding affordable childcare. In other words, they are worried about finding affordable childcare and thus, register their children to attend HAF. However, based on the current DfE HAF model of 4 h per day, for four days a week, and for four weeks during the summer school holiday, that some HAF clubs find challenging to deliver, parents may still be concerned about finding additional childcare throughout the school holidays. A further explanation may be that when answering the question about affordable childcare, this triggers a mental model of paid childcare. In which case, parents may be answering the questions with respect to childcare options that exclude HAF.

The current paper also found no differences between HAF attendees and non-attendees in terms of safe places for children to play within their local communities. Prior research studies [e.g., (12, 29)] found that parents perceived that HAF clubs provide a safe and secure environment for their children to play. It is important to point out that our findings do not suggest that HAF clubs do not provide a safe place for children and young people to play, rather the findings show no difference between groups regarding parent’s perceptions about the safety of their children playing in their local neighborhood. Of course, the presence of HAF clubs across the last few years, community organizing, and activity could result in a more general place-based improvement in perceptions of child safety, although we currently have no data to support this.

There is clear evidence, from across multiple studies, that HAF attendance increases children’s and young people’s participation in physical activity (2, 12, 29). This is particularly encouraging as research has shown that children’s participation in physical activity is important for both mental and physical health, cognition, and preventing childhood obesity (e.g., (51)). The findings of the current paper show that participation in physical activity is increased in the HAF group compared to the control group but only for those children who attend four weeks or more. Thus, suggesting the minimum 4 × 4 × 4 (4 h per day, 4 days per week, and 4 weeks) HAF delivery model, as originally proposed by Defeyter to the DfE, is sufficient in terms of increasing children’s physical activity.

As in the case of prior studies, the present study found that children’s HAF attendance improved parental wellbeing (59), but only for those parents whose children attended HAF for 6 weeks or more. Several research studies have shown that some parents report that school holidays are stressful because of additional financial obligations (e.g., (12)) and that HAF eases the financial burden on households during the school holidays (although see (21)). An increasing number of studies have found an association between food insecurity and poor mental health (9, 11). However, the findings of the current paper show no differences between groups in terms of household food insecurity, suggesting that other factors are driving improved self-reported mental wellbeing in HAF attendees’ parents compared to non-attendees. One possibility may be that the nutritional knowledge disseminated via HAF clubs improves dietary intake in both children and parents and this improved dietary intake improves parental wellbeing (52, 53); although see Round et al. (54). This idea requires further research to (a) ascertain whether the nutritional knowledge disseminated via HAF improves dietary intake and (b) whether it is associated with improved mental wellbeing. Alternatively, it may be the case that HAF increases or improves the quality of social contact and sense of community, provides parents and caregivers with some respite, and this increases wellbeing (12, 43).

In conclusion, this paper addresses some of the gaps identified in prior studies, e.g., a lack of a comparator (non HAF attendee) group. It also provides detailed quantitative analyses to complement many studies that have adopted a qualitative approach to exploring the efficacy and impact of HAF. Importantly, it provides the first reported findings on the associations between time attending HAF and health and wellbeing outcome measures.

However, conducting quantitative research with economically vulnerable populations can prove challenging as they are especially likely to ignore requests for information due to time constraints, limited resources, and high levels of mistrust (55). Researchers studying the potential benefits of Holiday Activities and Food (HAF) programs face these same challenges, as HAF participants often come from economically marginalized backgrounds and often must be accessed through resource strained service organizations (43).

In addition to these typical research obstacles, HAF researchers need to contend with the significant heterogeneity of HAF programs. For example, a typical minimum HAF enrolment during the summer holidays may follow a “4x4x4” service model, where children attend HAF for 4 h a day, 4 days a week, over 4 weeks (43). Still, some HAF providers exceed this minimum, offering more hours, days, and weeks of service. Despite the difficulties of accessing parents and caregivers whose children attend or could attend HAF we were able to explore the potential outcomes of HAF in one large UK local authority by utilizing a relatively simple, but efficient, correlational type of study design.

We suggest that there are two main limitations of the current study. Firstly, the findings are based on HAF attendance in one local authority in the West Midlands. One limitation of our sample strategy is that we are limited to one local authority area and therefore our results concerning HAF cannot be generalized to other local authorities or the entire HAF program across England as we know that HAF delivery models vary (26). Secondly, without a pre-and-post HAF intervention design, with an appropriate comparator group, it is not possible to demonstrate causality. While we controlled for differences between HAF Attendees and Non-Attendees groups in our analysis, it is clear that there are significant differences between groups, and future studies should carefully consider this point.

However, despite these limitations, we suggest that the findings of the current paper provide evidence for several policy recommendations. First, the increased parental mental wellbeing outcome and children’s increased engagement in physical activity, compared to non-attendees, demonstrated in the current study are, by themselves, strong reasons for the UK government to fund HAF beyond March 2025. Second, the finding that different levels of HAF attendance are associated with different outcomes suggests that the current 4 × 4 × 4 national HAF model requires further exploration, and possible extension, to maximize the programs impact across multiple outcomes. Finally, we recommend that, given the sharp increase in the number of children living in poverty (56), the UK government conducts an analysis on the costs and benefits that would be incurred in expanding HAF to (a) all families in receipt of Universal Credit and (b) to all children and young people living in areas of high multiple deprivation through adopting a targeted, universal approach that enables all children in these neighborhoods to attend HAF; akin to the UK Government’s Family Hub program.

## Data Availability

The raw data supporting the conclusions of this article will be made available by the authors, without undue reservation.

## Footnotes

^1^ www.prolific.com
