# Supplementary material for: A study comparing positive benefits for parents, and their children, of children attending the UK’s holiday activities and food program to parents of non-attendees
Source: Front Public Health. 2025 Feb 25;13:1474400. doi: 10.3389/fpubh.2025.1474400 (PMC11893578; doi:10.3389/fpubh.2025.1474400)
Supplement: Supplementary file 1 [file Data_Sheet_1.pdf]

Appendix A. Mean, Median, Range and Standard Deviation for Study Variables

	Range	Mean	Median	Std. Dev.
Outcome Variables				
Physical Exercise (6=More Exercise)	0-6	2.96	3.00	2.00
Household Food Security (6=Food Secure)	0-6	3.47	4.00	2.37
Affordable Childcare (10=More Affordable)	0-10	6.37	7.00	3.64
Parental Wellbeing (16 = Higher Wellbeing)	0-16	8.2	8.00	3.07
Perceptions of safety (5 = Children Safe)	45413	3.43	4.00	1.21
Indicator				
Didn't Attend HAF	0-1	0.56	0.00	0.50
1 Week or Less	0-1	0.05	0.00	0.22
2 Weeks	0-1	0.06	0.00	0.25
3 Weeks	0-1	0.07	0.00	0.26
4 Weeks	0-1	0.09	0.00	0.28
5 Weeks	0-1	0.06	0.00	0.23
6 Weeks or More	0-1	0.08	0.00	0.27
Household Characteristics				
Free school meals eligible (1=Yes)	0-1	0.66	1.00	0.48
Primary earner unemployed	0-1	0.10	0.00	0.29
*Single parent household (*1=Yes)	0-1	0.41	0.00	0.49
Household size	0-1	3.88	4.00	1.44
Respondent Characteristics				
Gender				
Male	0-1	0.15	0.00	0.40
Female	0-1	0.85	1.00	0.33
Age (in years)	18-70	36.98	36.00	7.14
Race / Ethnicity				
White English/British/Welsch/Irish/Scottish	0-1	0.61	1.00	0.49
Asian	0-1	0.18	0.00	0.38
Black	0-1	0.08	0.00	0.27
Other Ethnic Group	0-1	0.04	0.00	0.18
Mixed Ethnicity	0-1	0.06	0.00	0.23
Year				
2022	0-1	0.54	1.00	0.50
2021	0-1	0.46	0.00	0.50

Appendix B. Ordinal Logistic Regression Exploring the Relationship Between HAF Attendance (2021/2022) and Physical Exercise, Household Food Insecurity, Affordable Childcare, Parental Wellbeing and Perceptions of Safety

	<u>Model 1</u><br>Physical Exercise<br>95% CI<br>b (LL,UL)	<u>Model 2</u><br>Household Food Security<br>95% CI<br>b(LL,UL)	<u>Model 3</u><br>Affordable Childcare<br>95% CI<br>b(LL,UL)	<u>Model 4</u><br>Parental Wellbeing<br>95% CI<br>b(LL,UL)	<u>Model 5</u><br>Perceptions of Safety<br>95% CI<br>b(LL,UL)
Time Spent in HAF<br>(vs. Didn't Attend)					
1 Week or Less	-0.16(-0.61,0.28)	0.23(-0.23,0.69)	-0.01(-0.51,0.50)	0.42(-0.02,0.87)	0.23(-0.24,0.70)
2 Weeks	0.20(-0.20,0.59)	-0.02(-0.43,0.39)	-0.11(-0.58,0.36)	0.33(-0.06,0.72)	-0.09(-0.51,0.32)
3 Weeks	0.35(-0.02,0.72)	-0.01(-0.39,0.36)	0.09(-0.34,0.51)	0.26(-0.11,0.63)	-0.11(-0.50,0.28)
4 Weeks	**0.95(0.59,1.31)***	-0.45(-0.81,-0.08)	-0.31(-0.72,0.10)	0.32(-0.04,0.68)	-0.11(-0.48,0.27)
5 Weeks	**1.06(0.63,1.50)***	0.04(-0.40,0.48)	-0.14(-0.62,0.34)	0.35(-0.08,0.77)	0.26(-0.19,0.71)
6 Weeks or More	**1.79(1.41,2.18)***	-0.28(-0.65,0.10)	**-0.60(-1.02,-0.19)***	**0.64(0.27,1.01)***	0.20(-0.19,0.58)
n	1453	1455	1008	1453	1423
Test of parallel lines	sig	ns	sig	sig	sig
Chi-Square in<br>-2 Log Likelihood (17 df)	157.50	116.02	4019.52	566.70	4877.70
Cox & Snell Pseudo<br>R-Square	0.10	0.11	0.03	0.04	0.01

Note: All models control for (1) Free School Meals, (2) Primary Earner Unemployed, (3) Single Parent Household, (4) Household Size, (5) Gender, (6) Age, (8) Race/Ethnicity and (9) Year. Bolded (*) results for time spent in HAF are "statistically significant" under the Bonferroni Correction or $p < 0.008$ (i.e., $\alpha = 0.05 / 6$). Coefficients represent the increase (or decrease) in the likelihood of being in a higher category of the outcome variable when in a treatment group compared to the control group.

Appendix C. Summary of Means for non-HAF Control Group and HAF Treatment Groups Across Each Outcome Examined in Tables 2 to 6

	Physical Activity	Household Food Insecurity	Affordable Child Care	Parental Well Being	Perceptions of Safety	Sample Size
	Mean	Mean	Mean	Mean	Mean	n
Non-HAF	2.84	3.50	6.49	7.89	3.46	908
1 Week or Less	2.52	4.04	6.91	8.84	3.49	79
2 Weeks	2.64	3.75	6.55	8.70	3.22	101
3 Weeks	2.68	3.35	6.69	8.35	3.35	118
4 Weeks	**3.37***	3.14	5.99	8.53	3.30	135
5 Weeks	**3.53***	3.48	6.45	8.46	3.54	87
6 Weeks or More	**4.26***	3.26	**5.19***	**9.05***	3.57	123

Bolded (*) values indicate statistically significant differences between the HAF treatment group and the non-HAF control group, as identified in Tables 2 to 6. Tests of significance were corrected using the Bonferroni method to account for multiple comparisons.
